# Effectiveness of the Lilly Connected Care Program in Improving Glycemic Management Among Patients With Type 2 Diabetes in China: Retrospective Real-world Study

**DOI:** 10.2196/38680

**Published:** 2023-04-25

**Authors:** Benli Su, Yu Chen, Xingping Shen, Jianchao Guo, Yuchen Ding, Xiao Ma, Yuxin Yang, Dongfang Liu

**Affiliations:** 1 Department of Endocrinology The Second Hospital of Dalian Medical University Dalian China; 2 Department of Endocrinology The Second Hospital of Tianjin Medical University Tianjin China; 3 Department of Endocrinology Zhongshan Hospital of Xiamen University School of Medicine, Xiamen University Xiamen China; 4 Lilly Suzhou Pharmaceutical Co Ltd Shanghai China; 5 Department of Endocrinology The Second Affiliated Hospital of Chongqing Medical University Chongqing China

**Keywords:** type 2 diabetes mellitus, T2DM, diabetes management, Lilly Connected Care Program, LCCP, hemoglobin A_1c_, real-word study

## Abstract

**Background:**

Type 2 diabetes mellitus (T2DM) is a worldwide public health concern. Mobile health management platforms could be a potential way to achieve effective glycemic control.

**Objective:**

This study aimed to evaluate the real-world effectiveness of the Lilly Connected Care Program (LCCP) platform in glycemic control among patients with T2DM in China.

**Methods:**

This retrospective study included Chinese patients with T2DM (aged ≥18 years) from April 1, 2017, to January 31, 2020, for the LCCP group and from January 1, 2015, to January 31, 2020, for the non-LCCP group. Propensity score matching was used to match the LCCP and non-LCCP groups to reduce confounding, with covariates including age, sex, the duration of diabetes, baseline hemoglobin A_1c_ (HbA_1c_), and the number of oral antidiabetic medication classes. HbA_1c_ reduction over 4 months, the proportions of patients achieving an HbA_1c_ reduction of ≥0.5% or ≥1%, and the proportions of patients reaching to target HbA_1c_ level of ≤6.5% or <7% were compared between the LCCP and non-LCCP groups. Multivariate linear regression was used to assess factors associated with HbA_1c_ reduction.

**Results:**

A total of 923 patients were included, among whom 303 pairs of patients were well matched after propensity score matching. HbA_1c_ reduction during the 4-month follow-up was significantly larger in the LCCP group than the non-LCCP group (mean 2.21%, SD 2.37% vs mean 1.65%, SD 2.29%; *P*=.003). The LCCP group had a higher proportion of patients with an HbA_1c_ reduction of ≥1% (209/303, 69% vs 174/303, 57.4%; *P*=.003) and ≥0.5% (229/303, 75.6% vs 206/303, 68%; *P*=.04). The proportions of patients reaching the target HbA_1c_ level of ≤6.5% were significantly different between the LCCP and non-LCCP groups (88/303, 29% vs 61/303, 20.1%; *P*=.01), whereas the difference in the proportions of patients reaching the target HbA_1c_ level of <7% was not statistically significant (LCCP vs non-LCCP: 128/303, 42.2% vs 109/303, 36%; *P*=.11). LCCP participation and higher baseline HbA_1c_ were associated with a larger HbA_1c_ reduction, whereas older age, longer diabetes duration, and higher baseline dose of premixed insulin analogue were associated with a smaller HbA_1c_ reduction.

**Conclusions:**

The LCCP mobile platform was effective in glycemic control among patients with T2DM in China in the real world.

## Introduction

The prevalence of diabetes increased globally over the past decades, and diabetes has become a major public health concern [1,2]. The global economic burden of diabetes was estimated to increase from US $1.3 trillion in 2015 to US $2.1 trillion by 2030 [3,4]. In China, the prevalence of diabetes has sharply increased from less than 1% in the 1980s to 12.4% in 2018 [5,6].

Type 2 diabetes mellitus (T2DM) has been reported to be associated with a higher risk of cardiovascular death compared to the population without diabetes [7]. Considering the substantial and growing burden of T2DM, there is an urgent need for more effective approaches to glycemic control management. It has been widely recognized that self-management and education are the cornerstones of T2DM management [8]. The American Diabetes Association issued the standards of medical care in diabetes in 2022, emphasizing diabetes self-management education and support and recommending the application of diabetes technology for self-monitoring and self-management [9,10]. The guideline for the prevention and treatment of T2DM in China has also underlined education and self-management and provided detailed suggestions accordingly, including group or individual education and distance education [11]. However, patients often encounter various barriers in adhering to self-management programs due to a lack of knowledge about self-care activities, a lack of individualized and coordinated care, inconvenient and costly education sessions, and suboptimal patient-provider communication [12-14].

Mobile health (mHealth) refers to the monitoring and sharing of health information through cell phones or smartphones, handheld tablets, and other wireless devices, which is a potential way to overcome barriers such as distance to care, limited access, and the short supply of qualified diabetes educators [15]. Prior studies revealed that diabetes management apps are effective in glycemic control in patients with T2DM [16,17]. Additionally, diabetes education plays an important role in improving glycemic control and self-management behaviors in patients with T2DM [18]. The Lilly Connected Care Program (LCCP), officially launched in April 2017, is a national diabetes care and support program embedded in China’s largest social app called WeChat. A total of 80,000 patients have registered in the LCCP. Patients can view their blood glucose (BG) readings in their WeChat accounts and receive customized diabetes education, individualized system-based reminders, family support through a family portal function, physician intervention, and call center support out of hospital [19,20]. The content of the prerecorded educational courses, which are evidence based and include diet and exercise advice, are reviewed by Lilly Medical and external experts and then unified and personalized for patients via the WeChat-based service account, or users can search for topics of interest on the platform to learn by themselves. BG-testing results can be synchronized automatically to patients’ WeChat accounts for viewing, and patients are engaged in diabetes education, which includes exercise and dietary guidance on an as-need basis. Moreover, the LCCP can also provide the patient support model with individualized system-based reminders, self-management support, physician intervention, and call center support [19]. Thus, the LCCP is not only a device for BG monitoring but also an mHealth technology providing individualized and holistic self-management support to patients in China. Prior studies have demonstrated the effectiveness of the diabetes education within the LCCP in improving glycemic control in patients with T2DM using a single-arm design [20,21]; however, the comparative effectiveness of the overall LCCP versus not using the LCCP in glycemic control remains unknown.

Here, we conducted a multicenter, retrospective, and observational study that matched patients with T2DM enrolled in the LCCP with those not using the LCCP to evaluate the effectiveness of the LCCP in glycemic control among patients with T2DM in real-world settings. The primary objective was to examine the hemoglobin A_1c_ (HbA_1c_) reduction relative to baseline in patients with T2DM who actively used the LCCP (the LCCP group) and those not using the LCCP (the non-LCCP group). The secondary objective was to compare the proportions of patients who achieved a target glycemic response, defined as an HbA_1c_ reduction of ≥0.5% or ≥1%, and the proportions of patients who achieved the targeted HbA_1c_ level of <7% or ≤6.5% between the LCCP and non-LCCP groups.

## Methods

### Study Design and Participants

This was a multicenter, retrospective, and observational study conducted in China. Outpatients with T2DM who had inadequate glycemic control with oral antidiabetic medications and received premixed insulin analogue treatment were encouraged by their physicians to register on the LCCP platform without any financial incentives. The LCCP program was initiated in 2017, and our study included patients with T2DM who were recruited in the LCCP group from April 1, 2017, to January 31, 2020. We selected a representative real-world sample of patients with T2DM who had inadequate glycemic control on oral antidiabetic medications and received premixed insulin analogue treatment but were not enrolled in the LCCP as the control group. Patients in the non-LCCP group were those who visited the hospitals in 4 large cities across China, including Chongqing, Dalian, Tianjin, and Xiamen, from January 1, 2015, to January 31, 2020. The reason that the time window for the non-LCCP group identification was 2 years earlier than that for the LCCP group was to ensure an adequate sample size for propensity score matching (PSM). Both the LCCP and non-LCCP groups received their routine treatment for diabetes, which includes lifestyle changes and treatment with oral diabetic medications in addition to premixed insulin according to the treatment guidelines in China. During the study period, the LCCP and treatment landscape for patients with T2DM initiating insulin therapies in China remained largely unchanged.

Deidentified patient-level data were retrieved from WeChat for the LCCP group and from the electronic medical records collected from the hospital information systems in the 4 hospitals for the non-LCCP group. Informed consent was obtained for the LCCP group and waived for the non-LCCP group as data were retrospectively collected. For the LCCP group, after informed consent was obtained, the BG results measured by the BG meter would be uploaded automatically via Bluetooth and collected together with the patient demographics and drug prescription information. For the non-LCCP group, BG data were collected in the laboratories and extracted from the hospital information systems. The date of the first prescription of any premixed insulin analogue was defined as the “index date” in our analyses.

Our study inclusion criteria for the LCCP and non-LCCP groups were (1) patients with T2DM aged ≥18 years old on the index date; (2) the first use of premixed insulin analogue was between April 1 2017, and January 31, 2020, for the LCCP group and between January 1, 2015, and January 31, 2020, for the non-LCCP group; (3) had at least one self-reported HbA_1c_ result (“baseline HbA_1c_”), and the report date (T_0_) was within 3 weeks after the index date for the LCCP group and between 2 months prior to and 1 month after the index date for the non-LCCP group—if multiple readings were available, the result closest to the index date was used; (4) baseline HbA_1c_ ≥7% and ≤15%; and (5) had at least one self-reported HbA_1c_ result (“follow-up HbA_1c_”), and the report date (T_1_) was within 2 to 6 months after the index date—if multiple readings were available, the result closest to 4 months after the index date was used. An additional inclusion criterion for the LCCP group was being an active user on the LCCP platform, defined based on the following criteria: (1) patients who started using the LCCP smart BG-testing device within the first 3 weeks after enrollment and were followed up for 12 weeks; (2) patients who completed ≥3 diabetes education courses per week; and (3) those who had ≥6 self-monitoring BG tests per week during the 12-week follow-up period.

### Sample Size Calculation and Power

A review of 27 studies summarized that HbA_1c_ reduction ranged from 0.16% to 4.2% in Asian patients with T2DM who initiated premixed insulin analogue, including Humalog premixed insulin [22]. Accordingly, we calculated that 176, 100, and 64 patients per group would yield a power of 80% to detect a difference of HbA_1c_ reduction at 0.3%, 0.4%, and 0.5%, respectively, assuming the SD of HbA_1c_ reduction was 1% and the 2-sided significance level was .05.

### Outcome Assessment

We first compared the HbA_1c_ reduction between the LCCP and non-LCCP groups. Then, we analyzed the percentages of patients who achieved the glycemic response of an HbA_1c_ reduction of ≥0.5% or ≥1% and the percentages of patients who achieved the target HbA_1c_ levels of <7% or ≤6.5% after 4 months of follow-up in the LCCP and non-LCCP groups, respectively.

### Statistical Analysis

PSM was used to reduce confounding. PSM is a widely used approach to accounting for multiple confounders in observational studies. In PSM, a matched population of treated and untreated participants is constructed based on the probabilities of receiving treatment. The underlying factors associated with treatment assignment are balanced through PSM by pairing each treated patient with one or more untreated patients that were roughly equally likely to have received the treatment [23-26]. The propensity score was calculated using a logistic regression model, and we applied the 1:1 matching with a caliper width equal to 0.2 of the SD of the logit of the propensity score. Age, sex, the duration of diabetes, baseline HbA_1c_, and the number of oral antidiabetic medication classes were included in the logistic regression model as covariates.

We presented the mean and SD for continuous variables with normal distribution and the median and IQR for continuous variables with skewed distributions. The categorical variables were presented as numerals and percentages. Statistical comparisons between the LCCP and non-LCCP groups were conducted using 2-tailed independent *t* test and Mann-Whitney *U* test (for mean and median, respectively) for continuous variables and chi-squared test or Fisher exact test for categorical variables. We performed full matching in the PSM model and inverse-probability of treatment weighting analysis as a sensitivity analysis to test the robustness of the comparison results. Full matching constructs strata consisting of either one treated subject and at least one control subject or one control subject and at least one treated subject [27].

Multivariable linear regression before matching was used to identify the variables associated with HbA_1c_ reduction. The backward stepwise selection method was used in the multivariable regression models. Variable screening and model adjustment were conducted according to the contribution of covariates in the model. Akaike information criterion value and adjusted *R*^2^ were the preferred evaluation indexes for variable screening.

All statistical analyses were performed using R (version 4.0.2; R Foundation for Statistical Computing) statistical software. A 2-sided *P* value <.05 was considered statistically significant.

### Ethics Approval

The study conformed to the Declaration of Helsinki principles and was approved by the ethics committee of the Second Affiliated Hospital, Chongqing Medical University (ethics number: 2020 Ethics Review(62)); Second Hospital of Dalian Medical University (ethics number: 2021 Ethics Review(155)); Second Hospital of Tianjin Medical University (ethics number: 2021 Ethics Review(028)); and Zhongshan Hospital of Xiamen University (ethics number: 2021 Ethics Review(084)).

## Results

### Baseline Characteristics

A total of 923 adult patients (aged ≥18 years) with T2DM receiving premixed insulin analogue were included in the analyses. Among these patients, 523 were in the LCCP group and 400 were in the non-LCCP group. The baseline characteristics before and after PSM are presented in Table 1. Before matching, the LCCP group had younger age (52.32 years vs 57.49 years), less prescription of oral antidiabetic medication classes, higher baseline HbA_1c_ level (9.82% vs 9.51%), and shorter diabetes duration (1.01 years vs 9.02 years) than the non-LCCP group. The difference in sex distribution between the 2 groups was not statistically significant (*P*=.93). After the 1:1 PSM, 303 pairs of patients were eligible for further analyses. After matching, the 2 groups were well balanced on age, sex, the number of oral antidiabetic medication classes, and baseline HbA_1c_.

**Table 1:** Patient characteristics in the Lilly Connected Care Program (LCCP) and non-LCCP groups before and after propensity score matching.

Variable and level		Before matching			After matching		
		LCCP (n=523)	Non-LCCP (n=400)	SMD^a^ (%)	LCCP (n=303)	Non-LCCP (n=303)	SMD (%)
Age (years), mean (SD)		52.32 (11.88)	57.49 (9.91)	47.2	56.02 (11.02)	56.19 (10.31)	1.6
**Sex, n (%)**				0.6			0.7
	Female	192 (36.7)	148 (37)		112 (37)	111 (36.6)	
	Male	331 (63.3)	252 (63)		191 (63)	192 (63.4)	
**Number of oral antidiabetic medication classes, n (%)**				60.6			7.4
	0	226 (43.2)	108 (27)		108 (35.6)	105 (34.7)	
	1	222 (42.4)	140 (35)		123 (40.6)	120 (39.6)	
	2	66 (12.6)	104 (26)		63 (20.8)	65 (21.5)	
	≥3	9 (1.7)	48 (12)		9 (3)	13 (4.3)	
Baseline HbA_1c_^b^ (%), mean (SD)		9.82 (1.88)	9.51 (1.83)	16.8	9.64 (1.80)	9.61 (1.91)	1.7
Diabetes duration (years), median (IQR)		1.01 (0.07-7.65)	9.02 (2.98-14.02)	69.3	5.02 (0.20-10.63)	7.16 (2.01-12.07)	12.6

^a^SMD: standardized mean difference.

^b^HbA_1c_: hemoglobin A_1c_.

### Effectiveness of the LCCP in Glycemic Control

Table 2 shows the comparison of HbA_1c_ changes over approximately 4-month follow-up between the matched LCCP and non-LCCP groups. Mean HbA_1c_ at the follow-up visit were 7.43% (SD 1.86%) and 7.95% (SD 1.85%) in the LCCP and non-LCCP groups, respectively. The LCCP group had a significantly larger HbA_1c_ reduction compared to the non-LCCP group (mean 2.21%, SD 2.37% vs mean 1.65%, SD 2.29%; *P*=.003).

We also compared the proportion of patients who achieved the glycemic response of an HbA_1c_ reduction of ≥0.5% or ≥1%. Overall, 209 (69%) out of 303 participants in the LCCP group had an HbA_1c_ reduction ≥1% at follow-up, which was significantly higher than that in the non-LCCP group (174/303, 57.4%; *P*=.003). Additionally, the proportion of patients having an HbA_1c_ reduction of ≥0.5% was significantly higher in the LCCP group than that in the non-LCCP group (229/303, 75.6% vs 206/303, 68%; *P*=.04).

Results showed that the LCCP group had a larger proportion of patients reaching the target HbA_1c_ levels of <7% and ≤6.5% than the non-LCCP group, and the difference was statistically significant for the target HbA_1c_ level of ≤6.5% (88/303, 29% vs 61/303, 20.1%; *P*=.01; see Table 2). Full matching and inverse-probability of treatment weighting analysis results indicated the robustness of the results (see Multimedia Appendix 1).

To further investigate the variables associated with HbA_1c_ reduction, a multivariate linear regression model was used (adjusted *R*^2^=0.445). Results showed that LCCP participation and higher baseline HbA_1c_ were associated with a larger HbA_1c_ reduction, whereas older age, longer diabetes duration, and higher baseline dose of premixed insulin analogue were associated with a smaller HbA_1c_ reduction. Adjusting for age, baseline HbA_1c_, sex, the duration of diabetes, and baseline premixed insulin dose, patients in the LCCP group had 0.492% (*P*=.002) more HbA_1c_ reduction relative to baseline than the non-LCCP group (see Table 3).

**Table 2:** Comparison of hemoglobin A_1c_ (HbA_1c_) reduction between the Lilly Connected Care Program (LCCP) and non-LCCP groups after matching.

Group	LCCP (n=303)	Non-LCCP (n=303)	*P* value
Follow-up HbA_1c_ (%), mean (SD)	7.43 (1.86)	7.95 (1.85)	<.001
HbA_1c_ reduction (%)^a^, mean (SD)	2.21 (2.37)	1.65 (2.29)	.003
HbA_1c_ reduction ≥0.5%, n (%)	229 (75.6)	206 (68)	.04
HbA_1c_ reduction ≥1%, n (%)	209 (69)	174 (57.4)	.003
Target HbA_1c_ ≤6.5%, n (%)	88 (29)	61 (20.1)	.01
Target HbA_1c_ <7%, n (%)	128 (42.2)	109 (36)	.11

^a^HbA_1c_ reduction is calculated as baseline HbA_1c_ minus follow-up HbA_1c_.

**Table 3:** Variables associated with hemoglobin A_1c_ (HbA_1c_) reduction.

Variables	Level (comparator vs reference)	Estimate (SE)	Statistic	*P* value
LCCP^a^ participation	LCCP vs non-LCCP	0.492 (0.159)	3.101	.002
Age	N/A^b^	–0.021 (0.006)	–3.236	.001
Sex	Male vs female	0.185 (0.140)	1.317	.19
Baseline HbA_1c_	N/A	0.800 (0.037)	21.431	<.001
Duration of diabetes	Long vs short^c^	–0.393 (0.155)	–2.537	.01
Baseline dose of premixed insulin analogue	N/A	–0.011 (0.005)	–2.235	.03

^a^LCCP: Lilly Connected Care Program.

^b^N/A: not applicable.

^c^Long is defined as a duration of diabetes ≥5 years, and short is defined as a duration of diabetes <5 years.

## Discussion

### Principal Findings

This was the first retrospective observational study demonstrating the effectiveness of the LCCP in glycemic control among patients with T2DM in China using real-world patients as comparators. We found that patients with T2DM in the LCCP group had a larger HbA_1c_ reduction compared to those in the non-LCCP group (mean 2.21%, SD 2.37% vs mean 1.65%, SD 2.29%; *P*=.003), and the LCCP group had a higher proportion of patients with an HbA_1c_ reduction of ≥1% (209/303, 69% vs 174/303, 57.4%; *P*=.003) and ≥0.5% (229/303, 75.6% vs 206/303, 68%; *P*=.04) compared to the non-LCCP group. Additionally, the proportions of patients reaching the target HbA_1c_ level of ≤6.5% were statistically significantly different between the LCCP and non-LCCP groups (88/303, 29% vs 61/303, 20.1%; *P*=.01).

Diabetes education is critical for improving patients’ self-management [8]. Mobile apps can receive and transmit information at any time and any place. With the popularity of digital mobile devices, the potential role of mobile-enabled apps in supporting patient self-management of diabetes has been widely investigated [28]. Kitsiou et al [29] reviewed the evidence of mHealth intervention for patients with diabetes and concluded that mHealth interventions represent a promising approach for the self-management of diabetes. A randomized controlled trial compared the efficacy of a smartphone-based, patient-centered diabetes care system (mDiabetes) with that of a paper logbook (pLogbook) for patients with T2DM and demonstrated a significantly greater reduction (0.35%, 95% CI 0.14-0.55) in HbA_1c_ levels in the mDiabetes group [30]. A systematic review analyzed 71 commercial apps available on the Apple App Store and 16 apps with peer-reviewed publications for diabetes self-management and found that mobile apps can play a viable role in diabetes management [31]. A web-based survey conducted in the web-based community of persons with diabetes found 145 diabetes apps reported by the patients as diabetes self-management that were positively associated with self-care behavior, which suggested that those apps can lead to improvement in lifestyle and glucose monitoring in patients with diabetes [32]. Evidence from several studies reported that self-monitoring of BG using different methods showed favorable clinical outcomes in terms of glycemic control even in patients with diabetes who are treated with insulin [33,34].

Prior studies have investigated the use of the LCCP and illustrated its effectiveness in glycemic control through a single-arm design [19-21]. Lin et al [19] found that the LCCP users had significant reduction in fasting BG (FBG) and postprandial glucose at week 12 compared with baseline. Zhang et al [21] explored the effectiveness of the LCCP app-based diabetes education in glycemic control, where patients recruited to the LCCP were classified into 3 groups according to the number of courses taken (0-4 courses, 5-29 courses, and ≥30 courses) and the change in BG was compared between the groups, and showed that a larger number of diabetes education courses taken was associated with lower FBG and postprandial glucose control after 12-week follow-up. In another study, Zhang et al [20] evaluated the effectiveness of the family portal function in the LCCP in glycemic control and self-management behavior improvement. Patients with T2DM in the LCCP were categorized into the family portal group and non–family portal group based on whether the patients’ family members were engaged in using the family portal in their LCCP accounts. After 12-week follow-up, the family group had significantly lower FBG and postprandial BG than the nonfamily group.

All the aforementioned studies were conducted in LCCP participants without comparing to patients not using the LCCP. In our study, we included patients not using the LCCP as the control group to provide further evidence of the effectiveness of the LCCP. Prior studies also illustrated the behavior improvement among LCCP users, such as the increased number of diabetes education courses taken and higher frequency of self-monitoring of BG [19,20]. We also used PSM to match the characteristics between 2 groups to control for potential confounders. The characteristics between the LCCP and non-LCCP groups were comparable after matching except diabetes duration, which remained significantly shorter for the LCCP group than the non-LCCP group even after matching; this could be explained by the inherent difference between these 2 groups.

Besides LCCP participation, other variables associated with HbA_1c_ reduction were identified, such as age, sex, baseline HbA_1c_, and the duration of diabetes. Prior studies reported that women and younger patients were more willing to be involved in health app use than men and older adults [35,36]. We did not observe a statistically significant association between sex and HbA_1c_ reduction in this study.

Several limitations of this study are noted as follows. First, the follow-up period in this study was approximately 4 months, which was relatively short to observe the outcomes in diabetes management. Therefore, the long-term effectiveness of the LCCP education platform shall be further investigated. Second, because of the retrospective study design, some variables including residence, socioeconomic status, educational level, and diabetes-related complications were not collected or controlled for, which could potentially lead to confounding. Thus, randomized control trials to further evaluate the effectiveness and future studies with a larger sample size and longer follow-up period are needed. Third, recall bias might occur because HbA_1c_ levels in the LCCP group were self-reported by the patients.

### Conclusions

This real-world study found that the LCCP platform was effective in improving glycemic control among patients with T2DM in China. The LCCP platform provides an effective way to enhance glycemic control, patient education, and self-management in patients with T2DM, especially during the COVID-19 pandemic.

## Appendix

Multimedia Appendix 1 Comparison of hemoglobin A_1c_ change between the Lilly Connected Care Program (LCCP) and the non-LCCP groups after full matching and inverse-probability of weighting analysis.

## Abbreviations

BG: blood glucose
FBG: fasting blood glucose
HbA_1c_: hemoglobin A_1c_
LCCP: Lilly Connected Care Program
mHealth: mobile health
PSM: propensity score matching
T2DM: type 2 diabetes mellitus
