# Supplementary material for: Effectiveness of the Lilly Connected Care Program in Improving Glycemic Management Among Patients With Type 2 Diabetes in China: Retrospective Real-world Study
Source: J Med Internet Res. 2023 Apr 25;25:e38680. doi: 10.2196/38680 (PMC10170357; doi:10.2196/38680)
Supplement: Multimedia Appendix 1 [file jmir_v25i1e38680_app1.docx]

|  | **Full Matching** | | |  | **IPTW Analysis** | | |
| --- | --- | --- | --- | --- | --- | --- | --- |
| **Group** | LCCP | Non-LCCP | P value |  | LCCP | Non-LCCP | P value |
| **N** | 523 | 400 |  |  | 1910 | 892.2 |  |
| **Follow-up HbA1c, %** | 7.41 (2.08) | 7.77 (1.70) | .014 |  | 7.36 (2.20) | 7.85 (1.74) | .001 |
| **HbA1c reduction, % ^*^** | 2.41 (2.62) | 1.86 (2.39) | .007 |  | 2.67 (2.76) | 1.85 (2.31) | <.001 |
| **HbA1c reduction ≥ 0.5%, n (%)** | 396.0 (75.7) | 286.4 (71.6) | .236 |  | 1470.4 (77.0) | 643.6 (72.1) | .118 |
| **HbA1c reduction ≥ 1%, n (%)** | 362.0 (69.2) | 239.3 (59.8) | .013 |  | 1362.2 (71.3) | 540.3 (60.6) | .002 |
| **Targeted HbA1c ≤ 6.5%, n (%)** | 175.0 (33.5) | 86.2 (21.5) | .002 |  | 726.0 (38.0) | 188.3 (21.1) | <.001 |
| **Targeted HbA1c < 7.0%, n (%)** | 251.0 (48.0) | 156.4 (39.1) | .028 |  | 1001.6 (52.4) | 332.2 (37.2) | <.001 |

***** HbA1c reduction was calculated as baseline HbA1c minus follow-up HbA1c.

LCCP: Lilly Connected Care Program; IPTW: inverse-probability of treatment weighting; HbA1c: Hemoglobin A1c.
